# Relationship between skeletal muscle mass loss and metabolic dysfunction-associated fatty liver disease among Chinese patients with metabolic dysregulation

**DOI:** 10.1590/1806-9282.20230963

**Published:** 2024-03-04

**Authors:** Miao Xu, Yi Lin, Naibin Yang, Jialin Li, Li Li, Huiqing Ding, Chengfu Xu

**Affiliations:** 1 Zhejiang University School of Medicine, The First Affiliated Hospital, Zhejiang Provincial Clinical Research Center for Digestive Diseases, Department of Gastroenterology – Hangzhou, China.; 2 The First Affiliated Hospital of Ningbo University, Department of Endocrinology and Metabolism – Ningbo, China.; 3 University of Nottingham, Faculty of Humanities and Social Sciences, Center for Health Economics – Ningbo, China.; 4 The First Affiliated Hospital of Ningbo University, Metabolic (Dysfunction)-Associated Fatty Liver Disease Research Center, Department of Hepatology – Ningbo, China.; 5 The First Affiliated Hospital of Ningbo University, Department of Obstetrics and Gynecology – Ningbo, China.

**Keywords:** Metabolic syndrome, Skeletal muscle, Fatty liver, Sarcopenia, China

## Abstract

**OBJECTIVE:**

The aim of this study was to explore the correlation between skeletal muscle content and the presence and severity of metabolic dysfunction-associated fatty liver disease in patients with metabolic dysregulation in China.

**METHODS:**

A cross-sectional study was conducted among patients from the endocrinology outpatient department at Ningbo First Hospital, in Ningbo, China, in April 2021. Adult patients with metabolic dysregulation who accepted FibroScan ultrasound were included in the study. However, those without clinical data on skeletal muscle mass were excluded. FibroScan ultrasound was used to noninvasively evaluate metabolic dysfunction-associated fatty liver disease. The controlled attenuation parameter was used as an evaluation index for the severity of liver steatosis. Bioelectrical impedance analysis was used to measure the skeletal muscle index.

**RESULTS:**

A total of 153 eligible patients with complete data were included in the final analysis. As the grading of liver steatosis intensifies, skeletal muscle index decreases (men: P_trend_<0.001, women: P_trend_=0.001), while body mass index, blood pressure, blood lipid, uric acid, aminotransferase, and homeostatic model assessment of insulin resistance increase (P_trend_<0.01). After adjusting for confounding factors, a negative association between skeletal muscle index and the presence of metabolic dysfunction-associated fatty liver disease was observed in men (OR=0.691, p=0.027) and women (OR=0.614, p=0.022). According to the receiver operating characteristic curve, the best cutoff values of skeletal muscle index for predicting the metabolic dysfunction-associated fatty liver disease presence were 40.37% for men (sensitivity, 87.5%; specificity, 61.5%) and 33.95% for women (sensitivity, 78.6%; specificity, 63.8%).

**CONCLUSION:**

Skeletal muscle mass loss among patients with metabolic dysregulation was positively associated with metabolic dysfunction-associated fatty liver disease severity in both sexes. The skeletal muscle index cutoff value could be used to predict metabolic dysfunction-associated fatty liver disease.

## INTRODUCTION

With the improvement of living standards worldwide, metabolic dysfunction-associated fatty liver disease (MAFLD) is increasingly common, with a global prevalence of 25% among the healthy population^1^. In China, the prevalence of MAFLD among healthy people in Gansu Province in 2015, Henan Province in 2017, and northeast China in 2018 was 21.03–35.28%^2, 3,4^. The prevalence of overweight/obesity, dyslipidemia, hypertension, and hyperglycemia is much higher in China^2^. Sarcopenia is defined as the progressive loss of skeletal muscle mass, strength, and function^5^. Studies have found that sarcopenia increases the risk of diabetes, dyslipidemia, and cardiometabolic diseases^6^, ^7^.

Obesity is an independent risk factor for MAFLD^8^. Weight management may be an effective method to maintain body weight and weight loss^9^, ^10^ for the prevention of MAFLD^11^. Besides obesity, sarcopenia has been reported to have strong correlations with non-alcoholic fatty liver disease (NAFLD) and progressive cirrhosis^12^. The prevalence of sarcopenia was 46.0% in the older Chinese generation (men: 71.3 years and women: 69.9 years)^13^. Aging, a sedentary lifestyle, and low body mass index (BMI) are directly associated with sarcopenia^14^. Evidence of sarcopenia among young people with metabolic dysregulation is unknown.

Most studies have focused on fat mass accumulation in obese individuals. However, muscle mass loss has not received sufficient attention from researchers and clinicians. To the best of our knowledge, no study has been conducted on the association between muscle mass level and the presence and severity of steatosis with MAFLD in China, especially among patients with metabolic dysregulation. Therefore, this study aimed to investigate the association between muscle mass loss and MAFLD in patients with metabolic dysregulation in Ningbo, China.

## METHODS

### Ethics

Ethical approval was obtained from the Research Ethics Committee of Ningbo First Hospital (2019-R057). Written informed consent was obtained from all patients.

### Study design and patients

A cross-sectional study was conducted among patients from the endocrinology outpatient department at Ningbo First Hospital in Ningbo, China, in April 2021. Patients suspected of metabolic dysregulation and willing to undergo FibroScan ultrasound (PRO, Echosens, France) examinations were recruited. The data for anthropometric measurements, liver steatosis levels, skeletal muscle mass, biochemical tests, and questionnaires were all obtained during this visit. The inclusion criteria were as follows: 1. patients aged 18–75 years; 2. visiting the endocrinology outpatient department at Ningbo First Hospital for the first time; and 3. being diagnosed with metabolic dysregulation and underwent FibroScan ultrasound examination. Those patients without data on SMI were excluded. The diagnostic criterion for metabolic dysregulation satisfied at least one of the following conditions^15^: (1) overweight/obesity (BMI≥23 kg/m^2^)^16^; (2) type 2 diabetes mellitus (T2DM, according to the American Diabetes Association criteria); and (3) the presence of metabolic syndrome^17^.

### Data collection and study variables

All patients with metabolic dysregulation, willing to participate in this study, were asked to complete questionnaires, including demographic information (e.g., age and sex), medical history [T2DM, hypertension, hypertriglyceridemia, and low level of high-density lipoprotein (HDL) cholesterol], and medication records. Using the standardized protocol, anthropometry was measured by well-trained nurses, and biochemical parameters were analyzed by the laboratory staff.

### Anthropometric measurements

The BMI (kg/m^2^) was calculated as weight (kg)/height^2^ (m^2^). Bodyweight was measured to the nearest 0.1 kg with light clothes using a calibrated automatic digital weight. Height was measured to the nearest 0.5 cm without shoes in the standing position using a height scale (HNH-318, Omron, Japan). Waist circumference (in cm) was measured to the nearest 0.5 cm at the mid-point between the lower rib and iliac crest using a 150-cm medical tape. Blood pressure was measured on the right or left arm using an electronic sphygmomanometer (HBP-1100U, Omron, Japan) in a seated position after a 10-min rest.

### Biomarker measurements

Glycosylated hemoglobin (HbA1c) (%) was analyzed using high-performance liquid chromatography (D-10 Hemoglobin Analyzer, Bio-Rad, USA). Fast plasma glucose (FPG), alanine aminotransferase (ALT), aspartate aminotransferase (AST), triglyceride (TG), total cholesterol (TC), HDL cholesterol, low-density lipoprotein cholesterol (LDL-C), and uric acid (UA) were assessed by enzymatic assays (AU5400, Beckman Coulter, USA). Homeostatic model assessment of insulin resistance (HOMA-IR) was estimated using the following formula: fasting insulin (U/mL)×fasting glucose (mmol/L)/22.5. All anthropometric and biochemical parameters were obtained on the same day after overnight fasting for 8–10 h.

### Liver steatosis examination

FibroScan was designed to perform liver stiffness measurement using vibration-controlled transient elastography (VCTE) as a noninvasive medical device. The controlled attenuation parameter (CAP, dB/m), which is the ultrasonic attenuation coefficient of the ultrasonic signals used during VCTE examination, is correlated with hepatic steatosis. The CAP assessment was performed by an experienced sonologist, according to the FibroScan instructions^18^. Liver steatosis grade was determined by the cutoff values of CAP according to previous reports as follows: a. CAP<233.5 dB/m denoted no steatosis (S0), b. 233.5≤CAP<268.5 dB/m denoted mild steatosis (S1), c. 268.5≤CAP<301.2 dB/m denoted moderate (S2), and d. CAP>301.2 dB/m denoted severe steatosis (S3)^19^.

### Skeletal muscle mass measurement

Bioelectrical impedance analysis (BIA) was used to examine impedance for each segment, including the four limbs and trunk, using the InBody 770 body composition analyzer (InBody, Seoul, Korea). Multi-frequency measurements were performed to estimate appendicular skeletal muscle mass (ASM). In this study, skeletal muscle index (SMI, %) was calculated using the following equation: ASM/body weight (kg).

### Statistical analysis

Continuous variables with normal and skewed distributions are presented as the mean (standard deviation) and median (interquartile range), respectively. Continuous variables were analyzed using one-way analysis of variance and the Kruskal–Wallis test. Categorical variables are presented as numbers (percentages) and compared using the chi-square test according to the degree of liver steatosis. Multivariate logistic regression models were used to examine the associations between SMI and the presence of MAFLD using four models: (1) unadjusted model; (2) adjusted for age; (3) adjusted for age, diastolic blood pressure (DBP), and FPG; and (4) adjusted for age, DBP, FPG, TG, TC, UA, and ALT. Receiver operating characteristic (ROC) curve analysis of SMI was used to calculate the cutoff value of SMI for predicting the development of MAFLD. The results were considered statistically significant at a two-tailed level of 0.05. Statistical analyses were performed using IBM SPSS Statistics version 26.0 for Windows.

## RESULTS

A total of 188 patients diagnosed with metabolic dysregulation who underwent FibroScan ultrasound examinations were included, while 35 patients without clinical data on SMI were excluded. Finally, 153 patients (52.9% men and 47.1% women) with a mean age of 41.9 years were included in this study.

Table 1 shows the clinical characteristics and laboratory test results stratified by liver steatosis level. Approximately 26.8%, 17.6%, and 35.9% of patients were diagnosed with mild, moderate, and severe MAFLD, respectively. Significantly increasing trends were found in BMI, SBP, DBP, TG, TC, LDL-C, UA, ALT, AST, and HOMA-IR across groups of liver steatosis levels. In contrast, only SMI declined in both sexes.

**Table 1.** Characteristics of the study participants stratified by liver steatosis grades.

The grade of liver steatosis						
	S0<5%	S1 5–33%	S2 34–66%	S3=67%	F/H/χ^2^ values	P_trend_
	(n=30)	(n=41)	(n=27)	(n=55)		
Male, n (%)	16 (53.3)	24 (58.8)	13 (48.1)	28 (50.9)	0.857^b^	0.848
Age, years	48.5 (34.5, 58.0)	46.0 (32.0, 55.0)	40.0 (34.0, 57.0)	34.0 (26.0, 45.0)*	9.155^a^	**0.027**
BMI, kg/m^2^	25.75 (23.43, 27.25)	27.1 (23.8, 29.65)	27.7 (26.4, 31.2)*	30.2 (27.5, 37.0)**	37.8^a^	**<0.001**
DBP, mmHg	71.17 (9.26)	80.53 (11.39)**	75.52 (9.40)	83.80 (12.17)**	17.004	**<0.001**
FPG, mmol/L	5.49 (4.92, 7.44)	6.59 (5.07, 8.99)	5.68 (4.98, 7.18)	6.06 (5.30, 8.49)	5.033^a^	0.169
HbA1c, %	6.2 (5.4, 7.0)	6.2 (5.5, 7.2)	5.85 (5.28, 7.55)	6.5 (5.5, 8.4)	3.949^a^	0.267
TG, mmol/L	0.98 (0.73, 1.21)	1.52 (1.13, 2.73)**	1.54 (0.99, 1.88)	1.99 (1.32, 2.77)**	23.275^a^	**<0.001**
TC, mmol/L	4.47 (1.10)	5.13 (1.31)	4.66 (1.20)	5.48 (1.25)**	7.559	**0.007**
UA, µmol/L	319.49 (80.74)	355.06 (75.35)	344.00 (122.90)	389.02 (97.45)*	7.674	**0.006**
ALT, U/L	19.0 (11.0, 22.0)	23.0 (16.0, 32.0)	25.5 (19.5, 33.25)	36.0 (24.5, 85.0)**	31.902^a^	**<0.001**
HOMA-IR	2.61 (2.21, 3.94)	5.33 (3.09, 7.51)*	5.06 (4.13, 6.52)	7.57 (5.43, 15.00)**	31.943^a^	**<0.001**
CAP, dB/m	209.5 (200.5, 224.0)	254.0 (243.0, 263.0)**	285.0 (276.0, 296.0)**	334.0 (318.5, 357.0)**	140.062^a^	**<0.001**
SMI, %						
Male	42.37 (2.63)	40.14 (3.27)	40.10 (3.05)	36.74 (4.04)**	24.355	**<0.001**
Female	35.86 (2.97)	34.31 (3.36)	32.46 (3.30)*	33.55 (3.45)*	12.607	**0.001**

Statistically significant values are denoted in bold.

*compared with S0 group, p<0.05;

**compared with S0 group, p<0.01;

^a^H values;

^b^χ^2^ value.

Liver steatosis grade was decided by the cutoff values of CAP; CAP<233.5 dB/m denoted no steatosis (S0), 233.5=CAP=268.5 dB/m denoted mild (S1), 268.5=CAP=301.2 dB/m denoted moderate (S2), and CAP>301.2 dB/m denoted severe steatosis (S3). BMI: body mass index; DBP: diastolic blood pressure; FPG: fast plasma glucose; HbA1c: glycosylated hemoglobin; TG: triglyceride; TC: total cholesterol; UA: uric acid; ALT: alanine aminotransferase; HOMA-IR: homeostatic model assessment of insulin resistance; CAP: controlled attenuation parameter; SMI: skeletal muscle index.

Multivariate logistic regression was used to investigate the relationship between SMI and the presence of MAFLD stratified by sex (Table 2). Positive associations were found in model 1 for both men [odds ratio (OR)=0.713, p=0.002] and women (OR=0.764, p=0.009). After controlling for age, DBP, FPG, TG, TC, UA, and ALT, SMI was still maintained in model 4 in both men (OR=0.691, p=0.027) and women (OR=0.614, p=0.022).

**Table 2.** Odds ratio and 95% confidence intervals of skeletal muscle index for the presence of metabolic dysfunction-associated fatty liver disease by sex.

Male			
	OR	95% CI	p-value
Model 1	0.713	0.576–0.884	**0.002**
Model 2	0.711	0.570–0.887	**0.003**
Model 3	0.713	0.538–0.947	**0.019**
Model 4	0.691	0.498–0.959	**0.027**
**Female**			
	**OR**	**95% CI**	**p-value**
Model 1	0.764	0.625–0.934	**0.009**
Model 2	0.749	0.592–0.946	**0.015**
Model 3	0.64	0.46–0.891	**0.008**
Model 4	0.614	0.404–0.934	**0.022**

Statistically significant values are denoted in bold. Model 1: unadjusted; Model 2: adjusted for age; Model 3: adjusted for age, DBP, and FPG; and Model 4: adjusted for age, DBP, FPG, TG, TC, UA, and ALT. SMI: skeletal muscle index; DBP: diastolic blood pressure; FPG: fast plasma glucose; TG: triglyceride; TC: total cholesterol; UA: uric acid; ALT: alanine aminotransferase.

**Figure 1.**
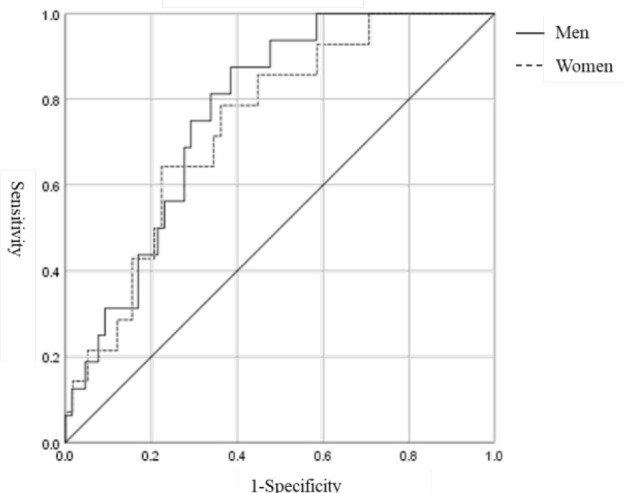
Receiver operating characteristic curves of skeletal muscle index to predict the presence of metabolic dysfunction-associated fatty liver disease by gender. Men (AUC=0.772, p=0.001), the optimal cutoff value was 40.37% with a sensitivity of 87.5% and a specificity of 61.5%; women (AUC=0.743, p=0.005), the optimal cutoff value was 33.95% with a sensitivity of 78.6% and a specificity of 63.8%.

The cutoff value of SMI for predicting MAFLD was analyzed using ROC curve analysis (Figure 1). The areas under the ROC curve were 0.772 [95% confidence interval (CI) 0.665–0.879, p=0.001] in men and 0.743 (95%CI 0.613–0.873, p=0.005) in women. The optimal cutoff values to predict MAFLD were 40.37% (with a sensitivity of 87.5% and a specificity of 61.5%) and 33.95% (with a sensitivity of 78.6% and a specificity of 63.8%) in men and women, respectively.

## DISCUSSION

This is the first cross-sectional study to examine the relationship between BIA-assessed skeletal muscle mass and liver steatosis in Chinese patients with metabolic dysregulation. We found that the loss of skeletal muscle mass was associated with the presence and severity of MAFLD in both men and women. The optimal cutoff values used to predict MAFLD were 40.37 and 33.95% in men and women, respectively.

There is a lack of information from research studies on the mechanism by which skeletal muscle works on abnormal fat accumulation in internal organs, especially MAFLD in patients with metabolic dysregulation. A previous meta-analysis, including 19 studies^20^, in line with our findings, indicated that the SMI level in patients with NAFLD was lower than that in healthy individuals. We found that MAFLD severity was positively associated with BMI, blood pressure, UA, HOMA-IR, TG, and TC. Insulin resistance might be the common pathogenesis of these metabolic disorders and MAFLD^21^ and promote the “first hit” of liver steatosis, characterized by hepatic TG accumulation^22^. Sarcopenic obesity has an increased risk of developing physical dysfunction compared to sarcopenia or obesity alone^23^. We hypothesized that skeletal muscle loss plays an important role in MAFLD occurrence and development. Skeletal muscle loss and intramuscular fat accumulation cause insulin resistance, oxidative stress, inflammatory cytokines, and mitochondrial dysfunction^24^. All of these factors may promote the “second hit” of MAFLD^22^. One myostatin secreted by skeletal muscle as an endocrine organ plays a role not only in regulating skeletal muscle mass and metabolism but also in liver steatosis^25^. In our study, after adjusting for confounders, including age, blood pressure, blood glucose, lipids, UA, and liver enzymes, we found that SMI was an independent protective factor for MAFLD in both men and women. Furthermore, the SMI cutoff value was estimated to predict MAFLD in approximately 40.37% of men and 33.95% of women.

### Limitations and strength

This is the first study to investigate the association between muscle mass and the presence and severity of steatosis with MAFLD among patients with metabolic dysregulation in China. However, this study had some limitations. First, the causal relationship could not be determined due to the cross-sectional study design. Second, the small sample size may have influenced the accuracy of this association. BIA and FibroScan are not the best methods for measuring body composition and liver steatosis, and golden standards should be used in the future.

## CONCLUSION

Skeletal muscle mass loss among people with metabolic dysregulation was positively associated with MAFLD severity in both sexes. The SMI cutoff value could be used to predict MAFLD. Furthermore, a prospective study with sufficient samples and intervention studies should be conducted to gain a deeper insight into the effect of skeletal muscle mass loss on MAFLD among patients with metabolic dysregulation.

## Data Availability

The dataset will be available upon request, unless there are legal or ethical reasons for not doing so.
